# Home blood pressure telemonitoring for improving blood pressure control in middle‐aged and elderly patients with hypertension

**DOI:** 10.1111/jch.14341

**Published:** 2021-08-05

**Authors:** Jianwei Yue, Xiaomin Yang, Bin Wang, Han Hu, Haiming Fu, Yuxia Gao, Gang Sun

**Affiliations:** ^1^ Department of Cardiology Tianjin Medical University General Hospital Tianjin P.R. China; ^2^ Research Institute of Hypertension Department of Cardiovascular Medicine The Second Affiliated Hospital of Baotou Medical College Baotou Inner Mongolia P.R. China; ^3^ Department of Clinical Laboratory Baotou Maternal and Child Health Center, Baotou, Inner Mongolia Autonomous Region P.R. China

**Keywords:** blood pressure monitoring, control, hypertension in the elderly, telemonitoring

## Abstract

The blood pressure (BP) control rate among treated hypertensives in China remains low at 37.5%. The relationship between home blood pressure telemonitoring (HBPT) and BP control is controversial. The authors aimed to investigate the relationship between HBPT and BP control in middle‐aged and elderly hypertensives. In total, 252 hypertension patients aged between 60 and 79 years were enrolled. The patients were given either HBPT through interactive platforms between physicians and patients (telemonitoring group, *n* = 126) or conventional management (routine management group, *n* = 126). All patients were followed‐up for 15 months. BP control was defined as home systolic blood pressure < 135 mm Hg and home diastolic blood pressure < 85 mm Hg. At baseline, there were no significant differences in the baseline BP control rate (*p *= .083). However, after 15 months, the BP control rate improved in both groups, and the telemonitoring group (71.3%) had a significantly higher BP control than the routine management group (49.8%) (*p *< .001). The change of BP control rate from baseline in the routine management group increased by 26.1%, and that of the telemonitoring group increased by 35.4%. The results of the fully adjusted binary logistic regression showed that HBPT was positively associated with BP control after adjusting for confounders (*OR* = 4.15, 95% *CI* 2.05–8.39). Similar results were observed after 3, 9, and 12 months. The association of HBPT with BP control was similar in subgroups. In conclusions, HBPT is recommended for BP control in middle‐aged and elderly hypertensives in the community setting.

## 1 INTRODUCTION

Hypertension is a primary risk factor for cardiovascular diseases,^1^, ^2^ and blood pressure (BP) control accordingly reduces the risk of cardiovascular disease and mortality.^3^, ^4^, ^5^ The standardized management rate of hypertension patients receiving treatment in China has reached 70.3%, but the control rate of hypertension is only 15.3%, and the treatment control rate is 37.6%.^6^ China has become an aging society, the data from 2015 Report on Nutrition and Chronic Diseases in China showed that hypertension was prevalent in 58.9% of the Chinese population aged older than 60 years in 2012.^7^ This highlights the urgent need for efficient management to improve the control rate of hypertension, especially in middle‐aged patients with hypertension.

Several studies have shown that home blood pressure telemonitoring (HBPT) is effective in lowering BP^8^, ^9^, ^10^, ^11^, ^12^ and reducing treatment costs.^13^, ^14^, ^15^ Accordingly, HBPT is recommended by an increasing number of experts.^16^, ^17^ However, the findings from previous studies regarding the relationship between HBPT and BP control are still controversial. In some randomized clinical trials, telemonitoring did not have a significant effect on improving BP control or lowering BP.^18^, ^19^, ^20^ In view of the differences in research design, target population, management method, and data analysis of these studies, additional studies are important. The effectiveness of HBPT in elderly patients has not been confirmed owing to the limitation of network operation technology in this population.^21^ Therefore, we aimed to investigate whether HBPT is independently related to BP control in middle‐aged and elderly patients with hypertension.

## 2 PARTICIPANTS AND METHODS

### 2.1 Study design

This was a nonrandomized controlled trial. The patients were assigned to different management groups at baseline. Patients in the telemonitoring group were given HBPT through interactive platforms between physicians and patients on the basis of routine management of hypertension. Meanwhile, the patients in the routine management group were given routine management of hypertension for a total of 15 months.

The target independent variable was the different methods of BP management, with or without HBPT. The dependent variable was BP control (dichotomous variable: 1 = BP control was defined as home systolic blood pressure (SBP) < 135 mm Hg and home diastolic blood pressure (DBP) < 85 mm Hg following 2019 Chinese Hypertension League guidelines on home blood pressure monitoring^22^; 0 = uncontrolled BP was defined as home SBP ≥135 mm Hg or home DBP ≥85 mm Hg).

This study was approved by the institutional review board of the Second Affiliated Hospital of Baotou Medical College (2017 Lun Shen No. 1). Written informed consent was obtained from each participant prior to the data collection. The study has been registered in the Chinese Clinical Trial Register, registration number: ChiCTR2100046153.

### 2.2 Study population

The participants were 252 hypertension patients aged 60–79 years (telemonitoring group, *n* = 126; routine management group, *n* = 126). They were recruited from seven communities in Baotou, China, between January 31, 2017 and July 31, 2017. Patients who were willing to participate in the program and who provided informed consent were included consecutively. Patients were grouped per their preferences; those who were willing to buy a Bluetooth sphygmomanometer and who consented to telemonitoring of home blood pressure were included in the telemonitoring group. Otherwise they were included in the routine management group. The inclusion criteria were as follows: (1) hypertension was diagnosed according to the Chinese guidelines for Prevention and treatment of Hypertension 2010,^23^ and (2) age 60–79 years. The exclusion criteria were (1) SBP ≥190 mm Hg or DBP < 60 mm Hg, (2) diagnosis of secondary hypertension, (3) atrial fibrillation and cancer, (4) poor control of diabetes (fasting blood glucose ≥11.1 mmol/L or HbA1c ≥8%), (5) severe liver or kidney disease (alanine aminotransferase ≥3 times higher than the normal upper limit; estimated glomerular filtration rate < 30 ml/min or serum creatinine > 2.5 ml [> 221 mmol/L] in dialysis patients), (6) cognitive impairment, or (7) inability for self‐care. Moreover, for patients in the telemonitoring group, we also required patients or their family members who live with them to own smartphones and have the ability to operate smartphones. Baseline data were obtained through a baseline questionnaire survey, and data were collected through Epidate double input.

### 2.3 Treatment protocol

Study physicians were assigned to each group to provide the assigned intervention. The patients in the two groups were managed by titration of antihypertensive treatment based on home BP measurements. The protocol for measuring home blood pressure for the two groups of patients was the same: BP was measured every morning and evening, with an emptied bladder, and measured after rest. The BP in every morning was measured within 1 h of waking up, prior to intake of antihypertensive drugs, and prior to eating breakfast. The BP at night was measured after dinner and before going to bed. Physicians prescribed individualized, guidance‐based medications for achieving normotension among the patients. Patients in the routine management group were followed‐up every 3 months. They were asked to self‐monitor the BP for 1 week before the outpatient follow‐up. They took their BP twice daily in the morning and evening and recorded their readings on paper. Their doctor titrated antihypertensive medications through these records. Patients in the routine management group received their own home automated upper arm sphygmomanometer certified by recognized international standard protocols (ESH, BHS, or AAMI).

Patients in the telemonitoring group measured their home BP using an automated BP device (HEM‐9200T, Omron Healthcare Co., Ltd)^24^ with Bluetooth transmission capability. They were asked to self‐monitor the BP twice in the morning and evening daily when the BP was uncontrolled. The interval was changed to 2 days a week after the BP was controlled. Both the patient and their doctor downloaded the “hypertension doctor” smartphone application (app). The smartphone app can be connected to the BP device via Bluetooth. The data are shared between the patient and the physician online.

The app has several key function: it can automatically remind participants to measure their BP; generate BP lists according to the measurement date and time; draw BP graphs; update the health knowledge regularly and send reminders to patients and doctors at three key points in time (when the red light is turned on once the BP exceeds the safe range; unplanned follow‐up reminders when the BP control rate does not reach 60% per month and the reminders of revisit time). Patients can communicate with the physician through the app, and the physicians can titrate the antihypertensive medicine for the patients in time. In addition, patients in telemonitoring group were followed‐up every 3 months. The physician also titrated the antihypertensive medicine in the outpatient follow‐up.

### 2.4 Follow‐up procedure

The outpatient follow‐up was carried out by the physician in charge of the management of hypertension patients, and the cutoff date for patient follow‐up was October 31, 2018. The follow‐up interval was 3 months. Monitoring indicators at each follow‐up included clinical BP and other physical examinations, and the doctor also titrated antihypertensive medicine and provided instructions on lifestyle based on the home BP.

### 2.5 Variables

HBPT was defined as a categorical variable. The final outcome variable (dichotomous variable) was BP control. Outcomes related to BP were based only on home blood pressure monitoring. Mean home blood pressure for the two groups of patients was calculated similarly. Home blood pressure was measured continuously for 7 days before each follow‐up, and the average of BP for the following 6 days was taken; the sum of all BP data was divided by the number of observations. BP control rate was calculated as the proportion of patients with controlled BP. These potential confounders were chosen on the basis of previous scientific literature, all significant covariates in the univariate analysis or a more than 10% change in effect estimates. Therefore, the following variables were used to build a fully adjusted model: (1) continuous variables: age, body mass index (BMI), duration of hypertension, creatinine, triglyceride (TG), high density lipoprotein cholesterol (HDL), baseline home systolic blood pressure (SBP), and diastolic blood pressure (DBP); and (2) categorical variables: sex, family history of hypertension, smoking status, and physical activity.

### 2.6 Statistical analysis

Continuous variables were expressed as the mean ± standard deviation, and categorical variables were expressed as frequencies or percentages. We used χ^2^ (categorical variables), Student's *t* test (normal distribution), or Mann‐Whitney U test (skewed distribution) to test for differences between the two groups. BP status (controlled or uncontrolled) was analyzed using univariate and multivariate binary logistic regression analyses. We constructed four models: model 1 was the unadjusted model; model 2 was only adjusted for age and sex; model 3, adjusted for covariates in model 2 plus BMI, duration of hypertension, smoking status, physical activity, and creatinine, TG, and HDL; model 4, adjusted for covariates in model 3 plus baseline home SBP and DBP. Subgroup analyses were performed using stratified binary logistic regression models. Age was first converted to a categorical variable according to a cutoff of 65 years, while duration of hypertension was adjusted according to a cutoff of 15 years. At 3, 6, 9, 12, and 15 months, general and generalized linear mixed models were used to test the effects of the intervention on SBP, DBP, and BP control outcomes. Time (categorical measure) and group were fitted as fixed effects while age, sex, BMI, duration of hypertension, smoking status, physical activity, creatinine, TG, and HDL were fitted as covariates. Interactions between time and group were considered so that the treatment effect could be assessed at each point in time. All analyses were performed using the statistical software packages R 3.3.2 (http://www.R‐project.org, The R Foundation), Free Statistics software version 1.3, and SPSS 26.0 (IBM, Armonk, NY, USA). Two‐tailed tests were also performed. Statistical significance was defined as a two‐sided *p* value < .05.

## 3 RESULTS

### 3.1 Baseline patient characteristics

In total, 116 and 136 patients were male and female, respectively. The mean age was 65.7 years (*SD*: 4.5, range: 60–79 years), and the mean BMI was 26.3 kg/m^2^ (*SD*: 3.0). The baseline patient characteristics stratified by group are presented in Table 1. The telemonitoring group was younger, had less physical activity, and had lower TG, but it also had a higher percentage of patients with a family history of hypertension. At baseline, the number of antihypertensive medications in the routine management group and the telemanagement group were 2 (1, 2) and 2 (1, 2), respectively (*p *= .294). In total, 125 patients in each group completed the 15‐month follow‐up (one patient in the telemonitoring group died of lung cancer, and one patient in the routine manager group withdrew due to an emerging tumor).

**TABLE 1:** Baseline characteristics of the study participants

	Total *N* = 252	Routine management group *N* = 126	Telemonitoring group *N* = 126	*p‐*value
Female/male	136/116	63/63	73/53	.255
Age, in years	65.7 ± 4.5	66.3 ± 4.5	65.1 ± 4.5	.031
Duration of HTN, in years	14.4 ± 9.3	13.6 ± 9.2	15.2 ± 9.4	.172
Diabetes	83 (32.9)	45 (35.7)	38 (30.2)	.421
Dyslipidemia	131 (52.0)	70 (55.6)	61 (48.4)	.313
Family history of HTN	172 (68.3)	75 (59.5)	97 (77)	.004
Physical activity	200 (79.4)	108 (85.7)	92 (73)	.020
smoking status	34 (13.5)	13 (10.3)	21 (16.7)	.197
BMI, in kg/m^2^	26.3 ± 3.0	26.3 ± 3.2	26.2 ± 2.8	.815
Cr, in μmol/L	73.3 ± 17.4	71.3 ± 17.7	75.3 ± 16.9	.066
UA, in mmol/L	345.6 ± 83.1	335.7 ± 86.7	355.5 ± 78.4	.059
FBG, in mmol/L	6.0 (5.3, 7.1)	6.0 (5.3, 7.2)	6.0 (5.4, 7.0)	.909
TG, in mmol/L	1.6 (1.1, 2.1)	1.7 (1.2, 2.4)	1.3 (1.0, 2.0)	< .001
CHO, in mmol/L	5.1 ± 1.2	5.0 ± 1.1	5.2 ± 1.3	.258
HDL, in mmol/L	1.4 ± 0.4	1.4 ± 0.4	1.3 ± 0.4	.292
LDL, in mmol/L	2.8 ± 0.9	2.7 ± 0.9	2.9 ± 1.0	.149
Baseline number of antihypertensive medications	2 (1, 2)	2 (1, 2)	2 (1, 2)	.294

*Note*: Data are mean ±SD, median (Q1–Q3), or *n* (%). Where relevant, some percentages might not add up to 100% due to rounding.

*Abbreviations*: BMI, body mass index; BP: blood pressure; CHO, total cholesterol; Cr, creatinine; DBP: diastolic blood pressure; FBG, fasting blood glucose; HDL, high density lipoprotein cholesterol; HTN, hypertension; LDL, low density lipoprotein cholesterol; SBP: systolic blood pressure; TG, triglyceride; UA, uric acid.

### 3.2 BP and BP control rate

At baseline, there was no significant difference in SBP (*p *= .167) (Table 2). After 15 months, mean SBP was lower in the telemonitoring groups (126.0 [95% *CI*, 124.3–127.6] mm Hg) compared with the routine management group (131.3 [95% *CI*, 129.6–132.9] mm Hg; *p *< .001). SBP of the routine management group decreased by 9.2 (95% *CI*, 6.6–11.8) mm Hg, and that of the telemanagement group decreased by 12.0(95% *CI*, 9.4–14.5) mm Hg. There was no significant difference in DBP between the two groups before and after management, except at 9 months. (*p *> .05).

**TABLE 2:** Blood pressure (BP) and BP reduction at 3, 6, 9, 12, and 15 months

	Routine management group		Telemonitoring group			
	Mean (95% *CI*)	Change From Baseline Mean (95% *CI*)	Mean (95% *CI*)	Change From Baseline Mean (95% *CI*)	Difference between the two group %(95% *CI*)	*P*
SBP (mm Hg)						
At baseline	140.5 (137.9,143.0)		137.9 (135.4,140.5)		−2.54 (‐6.13,1.06)	.167
At 3 month	134.8 (133.1,136.5)	−5.7 (‐8.3, ‐3.0)	128.1 (126.4,129.8)	−9.8 (‐12.5,‐7.2)	−6.71 (‐9.16,‐4.27)	<.001
At 6 month	132.0 (130.5,133.5)	−8.5 (‐10.9,‐6.0)	128.9 (127.4,130.4)	−9.0 (‐11.5,‐6.5)	−3.08 (‐5.21,‐0.95)	.005
At 9 month	130.4 (128.9,131.9)	−10.1 (‐12.6,‐7.6)	127.4 (125.9,128.9)	−10.5 (‐13.0,‐8.0)	−2.99 (‐5.15,‐0.84)	.007
At 12 month	130.4 (129.0,131.9)	−10.0 (‐12.5,‐7.6)	126.6 (125.2,128.1)	−11.3 (‐13.7,‐8.8)	−3.79 (‐5.84,‐1.74)	<.001
At 15 month	131.3 (129.6,132.9)	‐9.2 (‐11.8,‐6.6)	126.0 (124.3,127.6)	−12.0 (‐14.5,‐9.4)	−5.28 (‐7.63,‐2.94)	<.001
DBP (mm Hg)						
At baseline	83.8 (82.2,85.4)		84.1 (82.4,85.7)		0.28 (‐2.02,2.58)	.811
At 3 month	81.8 (80.5,83.1)	−2.0 (‐3.4,‐0.5)	80.7 (79.4,82.0)	−3.3 (‐4.7,‐1.9)	−1.09 (‐2.96,0.78)	.251
At 6 month	79.8 (78.5,81.1)	−4.0 (‐5.3,‐2.6)	80.9 (79.6,82.1)	−3.2 (‐4.5,‐1.8)	1.09 (‐0.69,2.87)	.228
At 9 month	78.1 (76.8,79.4)	−5.7 (‐7.1,‐4.3)	80.0 (78.7,81.3)	−4.0 (‐5.4,‐2.6)	1.95 (0.09,3.81)	.040
At 12 month	79.1 (77.9,80.4)	−4.7 (‐6.0,‐3.3)	79.9 (78.7,81.2)	−4.1 (‐5.5,‐2.8)	0.82 (‐0.93,2.57)	.357
At 15 month	79.1 (77.8,80.4)	−4.7 (‐6.1,‐3.3)	79.1 (77.8,80.4)	−5.0 (‐6.3,‐3.6)	0.02 (‐1.80,1.85)	.980

*Abbreviations*: BP, blood pressure; CI, confidence interval; DBP, diastolic BP; SBP, systolic BP.

At baseline, there was no significant difference in the BP control rate between the two groups (*p *= .083) (Table 3). With prolonged management, the BP control rate of patients in both groups gradually increased. After 15 months, BP was controlled in 71.3% (95% *CI*, 62.1%–80.5%) of the telemonitoring group and 49.8% (95% *CI*, 40.0%–59.6%) of the routine management group (*p *< .001). The change of BP control rate from baseline in the routine management group increased by 26.1% (95% *CI*, 11.2%–41.0%; *p *< .001), and that of the telemonitoring group increased by 35.4% (95% *CI*, 20.4%–50.5%; *p *< .001). The BP control rate at 3, 6, 12, and 15 months was significantly greater in the telemonitoring group than in the routine management group (*p* < .05).

**TABLE 3:** Blood pressure control rate at 3, 6, 9, 12, and 15 months

	Routine management group		Telemonitoring group			
	% (95% *CI*)	Change From Baseline % (95% *CI*)	% (95% *CI*)	Change From Baseline % (95% *CI*)	Difference between the two groups % (95% *CI*)	*p*
Baseline	23.7 (12.3,35.1)		35.9 (25.1,46.7)		12.2 (‐1.6,26.0(	.083
At 3 month	38.9 (28.3,49.4)	15.2 (0.1,30.5)	56.5 (46.5,66.5)	20.6 (5.0,36.3)	17.6 (5.2,30.1(	.005
At 6 month	49.3 (39.3,59.3)	25.6 (10.5,40.7)	61.3 (51.9,70.7)	25.4 (10.4,40.4)	12.0 (0.5,23.5(	.041
At 9 month	55.2 (45.2,65.1)	31.5 (16.3,46.7)	69.2 (59.9,78.6)	33.3 (18.2,48.5)	14.0 (2.7,25.4(	.016
At 12 month	55.6 (45.6,65.7)	31.9 (16.5,47.4)	68.1 (58.7,77.6)	32.2 (17.1,47.4)	12.5 (1.0,24.0(	.034
At 15 month	49.8 (40.0,59.6)	26.1 (11.2,41.0)	71.3 (62.1,80.5)	35.4 (20.4,50.5)	21.5 (10.4,32.7(	<.001

*Abbreviation*: CI: confidence interval.

During the 15 months of management, 68 additional follow‐ups were recorded, with 57 patients receiving an average of 1.2 additional follow‐ups per person. At 15 months, the number of antihypertensive medications in the routine management group and the telemanagement group were 2 (2, 2) and 2 (1, 3), respectively (*p *= .961).

### 3.3 Univariate analysis

Blood pressure control was correlated with the management methods. The results of the univariate analyses are listed in Table S1. HBPT, sex, age, duration of hypertension, physical activity, BMI, and Cr were found to be associated with achieving BP control.

### 3.4 Results of unadjusted and adjusted binary logistic regression

We constructed three models to analyze the independent effects of HBPT on BP control at 15 months (multivariate binary logistic regression). The effect sizes (odds ratio [*OR*]) and 95%*CIs* are listed in Table 4. After adjustment in multivariable analyses, HBPT were significantly associated with BP control. Compared with routine management, the addition of telemonitoring and timely intervention by a physician resulted in a higher BP control rate, with an *OR* of 4.15 (95% *CI* 2.05–8.39).

**TABLE 4:** Association between group and blood pressure control at 15 months

	*OR* (95% *CI*)	*p*‐value
Nonadjusted	2.36 (1.40,3.97)	.001
Adjust I	2.63 (1.50,4.61)	< .001
Adjust II	3.55 (1.81∼6.97)	< .001
Adjust III	4.15 (2.05∼8.39)	< .001

*Notes*: data presented are *OR*s and 95% *CI*s.

Adjust I model adjusts for age and sex.

Adjust II model adjusts for Adjust I factors + body mass index, duration of hypertension, family history of hypertension, physical activity, smoking status, creatinine, triglyceride, high density lipoprotein cholesterol. Adjust III model adjusts for Adjust II factors + baseline systolic blood pressure and baseline diastolic blood pressure.

We also constructed three models to analyze the independent effects of HBPT on BP control at 3, 6, 9, and 12 months (multivariate binary logistic regression). The results showed that HBPT was positively associated with BP control after adjusting for confounders at 3,9, and 12 months. The *OR* were 2.20 (95% *CI*, 1.20–4.03), 2.75 (95% *CI*: 1.41–5.37), and 2.62 (95% *CI*, 1.32–5.19), respectively (*p *< .05), (Table S2).

### 3.5 Subgroup analysis

**FIGURE 1:**
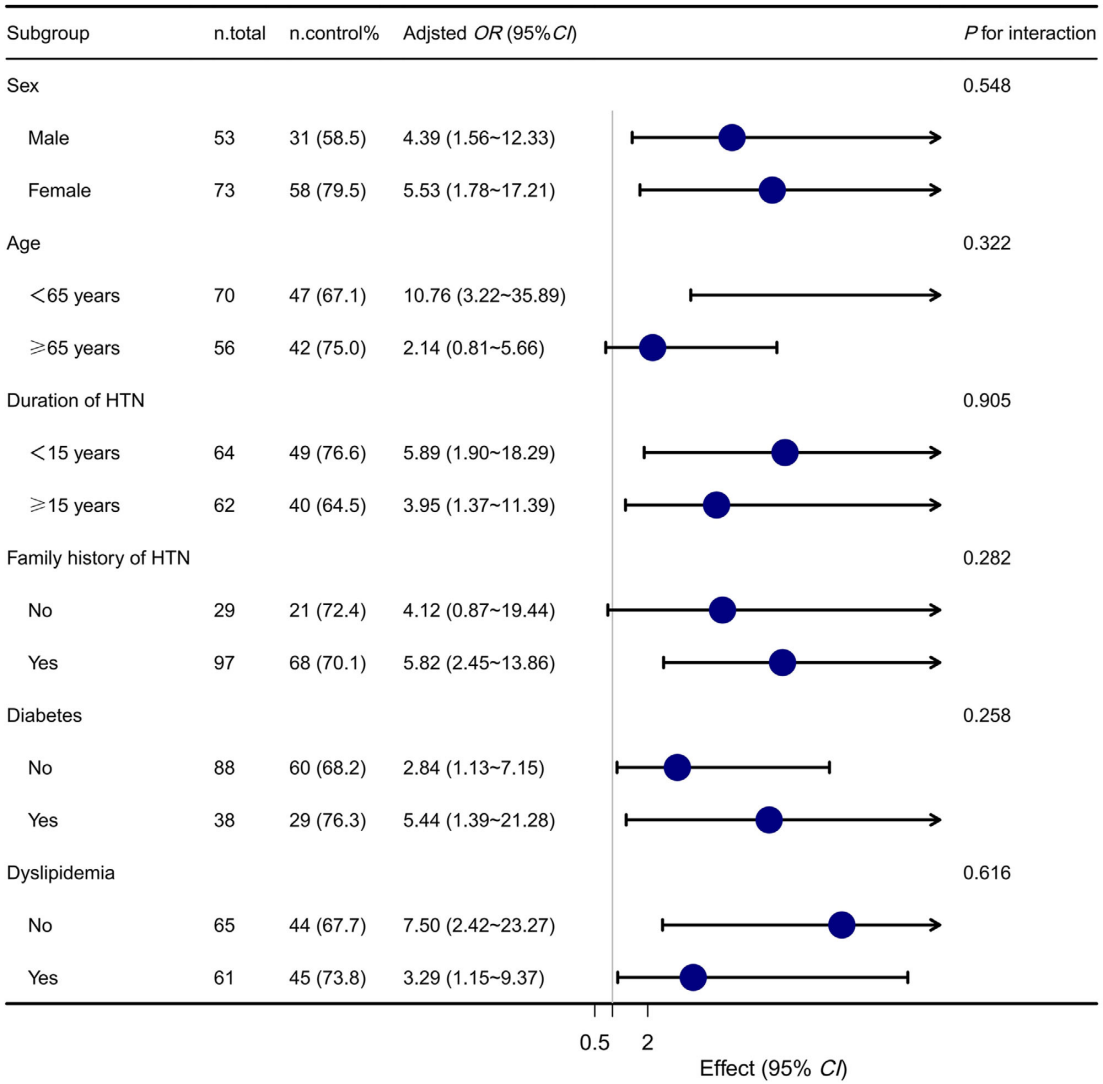
Forest plot for the subgroup analyses conducted to determine the size of the effect of using different types of BP management at 15 months following the interventions. Adjusted for age, sex, body mass index, duration of hypertension, family history of hypertension, smoking status, physical activity, creatinine, triglyceride, high density lipoprotein cholesterol, baseline systolic blood pressure, and baseline diastolic blood pressure. *Abbreviations*: n.total, total number of patients in the telemonitoring group; n.control%, the number of patients in the telemonitoring group whose blood pressure had been controlled

Stratified analyses of the associations between HBPT and BP control are presented in Figure 1. The effect sizes of HBPT on BP control in the subgroups were similar. The multiplicative interactions of BP control× sex (*p* for interaction = .548), BP control×age (*p* for interaction = .322), BP control×duration of hypertension (*p* for interaction = .905), BP control×family history of hypertension (*p* for interaction = .282), BP control×history of diabetes (*p* for interaction = .258), BP control×history of dyslipidemia (*p* for interaction = .616), and the OR of BP control were not significant. Some of the comparisons had wide *CI*s, reflecting relatively few individuals in some of the groups.

## 4 DISCUSSION

This study showed that the use of telemonitoring enabled timely intervention for the management of hypertension in middle‐aged and elderly patients and ultimately resulted in higher BP control rate. After 15 months, patients in the telemonitoring group had significantly lower systolic BP and higher BP control rate than those in the routine management group. Our findings indicate that HBPT is positively associated with BP control after adjusting for other covariates. Similar results were observed at 3, 9, 12, and 15 months. Subgroup analyses showed that there were no significant interactions between HBPT and age, sex, duration and family history of hypertension, diabetes or dyslipidemia with respect to BP control.

In the prospective observational cohort study by Ciemins and coworkers,^25^ 484 patients with uncontrolled hypertension were given BP monitors that transmitted the patients’ BP logs via Bluetooth or a USB cable to an iPhone, iPad, iPod Touch, or Android device. This system not only allowed patients to review their BP readings, but also allowed the patients’ team of nurses and clinicians to review the BP database and subsequently address critical BP values. The study showed that the improvement in BP control rates were significantly higher in the m‐health system group than that in the matched control group (from 42% to 67% vs. from 59% to 67%, *p *< .01). Margolis and coworkers^9^, ^10^ suggested that HBPT combined with pharmacist management reduced BP in diabetes patients with uncontrolled systolic hypertension and improved hypertension control. This management method had sustained effects for up to 24 months (12 months after the intervention ended).^9^ Chandler and coworkers^26^ also found that HBPT combined with electronic medication trays can also significantly reduce SBP among Hispanic adults with uncontrolled hypertension. Similar findings have also been reported in meta‐analyses.^27^, ^28^, ^29^ In 2017, a systematic review and meta‐analysis of 46 randomized controlled trials on home BP telemonitoring was published.^29^ These randomized studies included 13875 patients. A larger proportion of patients achieved BP normalization in the intervention group (relative risk [*RR*]: 1.16; 95% *CI*: 1.08–1.25; *p *< .001). Consistent findings were found in our study.

However, there are also some other studies that are inconsistent with our findings. The TASMINH4 study^8^ suggested that there is no significant difference between titration of antihypertensive medication guided by telemonitoring and only by home BP, but telemonitoring can reduce BP faster. This may be due to the following reasons. First, the methods of data transmission were different. In the TASMINH4 study, patients in the telemonitoring group sent their readings to a secure centralized database using a free SMS text message with web‐based data entry back up. In our study, BP data were automatically uploaded through a Bluetooth sphygmomanometer. Second, the frequency of BP titration was different. In their study, the BP was titrated once a month, and the home BP readings were recorded only 1 week every month in both intervention groups. However, in our study, the BP was titrated in time according to the three abnormal reminders of APP in the telemonitoring group, and the home BP data were measured twice a day or three times a week. Third, the study population was different. Participants were older than 35 years in the TASMINH4 study, whereas we evaluated patients aged 60–79 years. Finally, the BP data used for the analysis were different. They used a clinic BP reading of 6 or 12 months, whereas we used the mean home self‐measured BP at the 15th month.

Subgroup analyses of the Home and Online Management and Evaluation of BP trial showed that the size of the effect was smaller in participants aged 67 years or older (−0.4 mm Hg, 95% confidence interval −3.9 to 3.0 mm Hg) than in those aged 18–67 years (−7.7 mm Hg, −11.9 to −3.5 mm Hg).They reported that the treatment effect was much greater in patients aged 30–59 years, whereas it was generally equal in older patients in the intervention and usual care groups.^30^ However, subgroup analyses of the TASMINH4 trial^8^ suggested that there was no evidence of a differential effect in the subgroups examined, including by age (≥68 years and < 68 years). Our findings are consistent with the latter reports. The results show that HBPT improves BP control in individuals aged ≥60 years and that it is positively associated with BP control in both patients aged ≥65 years and < 65 years.

The clinical value of our study is as follows: (1) smartphone apps can provide patients with accurate medical information and provide tools to promote self‐monitoring and self‐management, (2) Our management methods are HBPT combined with educational support, danger reminders, doctor‐patient interaction, and real‐time intervention through smartphone apps. These methods may help optimize antihypertensive treatment, reduce the frequency of face‐to‐face consultations, and have the potential to facilitate patient self‐management, (3) Furthermore, it might be expected to offer particular advantages to patients in remote areas where telemonitoring might enable access to a physician, and (4) When patients’ BP exceeded the safe range, doctors provided patients with timely intervention, which greatly reduced the occurrence of hypertensive crisis. Therefore, we believe that telemonitoring and management can improve the long‐term prognosis of patients. Although there is still a lack of data in this regard, we will continue to observe for long‐term prognosis.

Meanwhile, the study limitations include potential selection bias due to a non‐randomized controlled design. However, we adjusted for confounders by incorporating covariates into the association analysis. The stability of the results was verified by subgroup analysis. In addition, our research participants were middle‐aged elderly patients with hypertension, but we excluded those who met the exclusion criteria, such as SBP ≥190 mm Hg or DBP < 60 mm Hg. These limitations limit the generalizability of our findings. Future studies should include patients aged younger than 60 years. We did not analyze patient adherence and could not exclude bias. However, we considered that adherence of the telemonitoring group may be better, and that the management mode of telemonitoring should also promote adherence. In future research, we will consider the impact of adherence.

In conclusions, HBPT helps improve BP control and lowers BP in middle‐aged and elderly patients with hypertension. HBPT is positively associated with BP control. HBPT provides remarkable support decision tools for physicians. Thus, HBPT can be recommended for the management of BP in the community setting in middle‐aged and elderly patients with hypertension.

## CONFLICT OF INTEREST

None

## AUTHOR CONTRIBUTIONS

Jianwei Yue, Xiaomin Yang, and BinWang contributed equally to this work. Correspondence: Yuxia Gao and Gang Sun. Gang Sun: conceptualization, supervision, project administration. Yuxia Gao: methodology, validation. Jianwei Yue: writing ‐ original draft, investigation, resources. Xiaomin Yang: writing ‐ review & editing, investigation. Bin Wang: visualization, writing ‐ review & editing. Han Hu: data curation, resources. Haiming Fu: resources, data curation.

## Supporting information

Supplementary information
