# Supplementary material for: Home blood pressure telemonitoring for improving blood pressure control in middle‐aged and elderly patients with hypertension
Source: J Clin Hypertens (Greenwich). 2021 Aug 5;23(9):1744–51. doi: 10.1111/jch.14341 (PMC8678721; doi:10.1111/jch.14341)
Supplement: Supplementary file 1 — Supplementary information [file JCH-23-1744-s001.docx]

| Supplemental Table 1 Univariate analysis for blood pressure control at 15 months | | | |
| --- | --- | --- | --- |
| Item | *OR* (95% *CI*) | *P* (Wald's test) |  |
| HBPT | 2.36 (1.40,3.97) | 0.001 |  |
| Man | 2.89 (1.71,4.89) | < 0.001 |  |
| Age | 1.08 (1.02,1.15) | 0.010 |  |
| Duration of HTN | 0.97 (0.94,1.00) | 0.026 |  |
| Diabetes | 0.91 (0.53,1.57) | 0.743 |  |
| Dyslipidemia | 1.44 (0.87,2.41) | 0.158 |  |
| Family history of HTN | 0.79 (0.45,1.37) | 0.398 |  |
| Physical activity | 0.45 (0.23,0.90) | 0.024 |  |
| Smoking status | 0.59 (0.28,1.22) | 0.152 |  |
| BMI, kg/m^2^ | 0.89 (0.82,0.98) | 0.013 |  |
| Cr, umol/l | 0.98 (0.97,1.00) | 0.032 |  |
| UA, mmol/l | 0.9976 (0.9945,1.0007) | 0.126 |  |
| FBG, mmol/l | 0.96 (0.83,1.12) | 0.633 |  |
| TG, mmol/l | 1.06 (0.88,1.27) | 0.546 |  |
| CHO, mmol/l | 1.11 (0.90,1.37) | 0.341 |  |
| HDL, mmol/l | 2.13 (0.98,4.65) | 0.057 |  |
| LDL, mmol/l | 1.12 (0.86,1.48) | 0.400 |  |
| Note: Data presented are *OR*s and 95% *CI*s.  Abbreviations: HTN, hypertension; BMI, body mass index; Cr, creatinine; UA, uric acid; FBG, fasting blood glucose; TG, triglyceride; CHO, total cholesterol; HDL, high density lipoprotein cholesterol; LDL, low density lipoprotein cholesterol | | | |

| Supplemental Table 2 Associations between groups and HTN control of BP at 3, 6, 9, and 12 months | | | | | | | | | | | | | | | |
| --- | --- | --- | --- | --- | --- | --- | --- | --- | --- | --- | --- | --- | --- | --- | --- |
|  | HTN control of BP at 3 months | |  | HTN control of BP at 6 months | |  | HTN control of BP at 9 months | | | |  | | HTN control of BP at 12 months | | |
|  | *OR* (95% *CI*) | *P-*value |  | *OR* (95% *CI*) | *P-*value | | |  | *OR* (95% *CI*) | *P-*value | |  | | *OR* (95% *CI*) | *P-*value |
| Nonadjusted | 1.96 (1.19,3.24) | 0.008 |  | 1.52 (0.92,2.51) | 0.100 | | |  | 1.72 (1.02,2.89) | 0.040 | |  | | 1.62 (0.96,2.71) | 0.068 |
| Adjust I | 2.03 (1.21,3.43) | 0.008 |  | 1.56 (0.93,2.62) | 0.091 | | |  | 1.75 (1.02,3.01) | 0.042 | |  | | 1.66 (0.96,2.87) | 0.072 |
| Adjust Ⅱ | 2.06 (1.15~3.7) | 0.016 |  | 1.51 (0.84~2.71) | 0.173 | | |  | 2.38 (1.26~4.49) | 0.008 | |  | | 2.17 (1.15~4.11) | 0.017 |
| Adjust Ⅲ | 2.20 (1.20~4.03) | 0.011 |  | 1.68 (0.89~3.15) | 0.107 | | |  | 2.75 (1.41~5.37) | 0.003 | |  | | 2.62 (1.32~5.19) | 0.006 |
| Notes: data presented are ORs and 95% CIs. | | | | | | | | | | | | | | | |
| Adjust I model adjusts for age and sex. | | | | | | | | | | | | | | | |
| Adjust Ⅱ model adjusts for Adjust I factors + body mass index, duration of hypertension, family history of hypertension, physical activity, creatinine, triglyceride, high density lipoprotein cholesterol, and smoking status. | | | | | | | | | | | | | | | |
| Adjust Ⅲ model adjusts for Adjust Ⅱ factors + baseline systolic blood pressure and baseline diastolic blood pressure.  Abbreviations: HTN, hypertension; BP, blood pressure; OR, odds ratio; CI; confidence interval. | | | | | | | | | | | | | | | |
